# Altered perivascular space diffusivity dynamics in motor neuron disease

**DOI:** 10.1093/braincomms/fcag307

**Published:** 2026-08-10

**Authors:** Ilaria Bottale, Edoardo G Spinelli, Silvia Basaia, Alma Ghirelli, Andrea Gardoni, Tommaso Russo, Elisa Canu, Veronica Castelnovo, Paride Schito, Yuri Falzone, Massimo Filippi, Federica Agosta

**Affiliations:** Neuroimaging Research Unit, Division of Neuroscience, IRCCS San Raffaele Scientific Institute, Milan 20132, Italy; Vita-Salute San Raffaele University, Milan 20132, Italy; Neurology Unit, IRCCS San Raffaele Scientific Institute, Milan 20132, Italy; Neuroimaging Research Unit, Division of Neuroscience, IRCCS San Raffaele Scientific Institute, Milan 20132, Italy; Vita-Salute San Raffaele University, Milan 20132, Italy; Neurology Unit, IRCCS San Raffaele Scientific Institute, Milan 20132, Italy; Neuroimaging Research Unit, Division of Neuroscience, IRCCS San Raffaele Scientific Institute, Milan 20132, Italy; Neuroimaging Research Unit, Division of Neuroscience, IRCCS San Raffaele Scientific Institute, Milan 20132, Italy; Vita-Salute San Raffaele University, Milan 20132, Italy; Neurology Unit, IRCCS San Raffaele Scientific Institute, Milan 20132, Italy; Neuroimaging Research Unit, Division of Neuroscience, IRCCS San Raffaele Scientific Institute, Milan 20132, Italy; Vita-Salute San Raffaele University, Milan 20132, Italy; Neurology Unit, IRCCS San Raffaele Scientific Institute, Milan 20132, Italy; Neuroimaging Research Unit, Division of Neuroscience, IRCCS San Raffaele Scientific Institute, Milan 20132, Italy; Neurology Unit, IRCCS San Raffaele Scientific Institute, Milan 20132, Italy; Neuroimaging Research Unit, Division of Neuroscience, IRCCS San Raffaele Scientific Institute, Milan 20132, Italy; Neurology Unit, IRCCS San Raffaele Scientific Institute, Milan 20132, Italy; Neurology Unit, IRCCS San Raffaele Scientific Institute, Milan 20132, Italy; Neuroimaging Research Unit, Division of Neuroscience, IRCCS San Raffaele Scientific Institute, Milan 20132, Italy; Vita-Salute San Raffaele University, Milan 20132, Italy; Neurology Unit, IRCCS San Raffaele Scientific Institute, Milan 20132, Italy; Neurorehabilitation Unit, IRCCS San Raffaele Scientific Institute, Milan 20132, Italy; Neurophysiology Service, IRCCS San Raffaele Scientific Institute, Milan 20132, Italy; Neuroimaging Research Unit, Division of Neuroscience, IRCCS San Raffaele Scientific Institute, Milan 20132, Italy; Vita-Salute San Raffaele University, Milan 20132, Italy; Neurology Unit, IRCCS San Raffaele Scientific Institute, Milan 20132, Italy

**Keywords:** glymphatic system, DTI-ALPS, amyotrophic lateral sclerosis, motor neuron disease, MRI

## Abstract

Converging evidence supports a key pathogenic role of the glymphatic system in the accumulation of pathological aggregates in several central nervous system proteinopathies, including amyotrophic lateral sclerosis and other motor neuron diseases. This study aimed to investigate potential glymphatic impairment using diffusion tensor imaging analysis along the perivascular space (DTI-ALPS) across motor neuron disease phenotypes, to examine its clinical correlates, and to assess its relationship with white matter damage.

Fifty-seven patients with motor neuron disease and 32 age- and sex-matched healthy controls underwent a 3 Tesla brain MRI scan, including diffusion tensor imaging sequences. We obtained the DTI-ALPS index from each individual, evaluating its relationship with measures of motor and cognitive disability, site of symptom onset, cognitive status, genetic status and fractional anisotropy of white matter tracts. Comparisons between groups were evaluated using analysis of covariance adjusting for age, sex, local fractional anisotropy and white matter hyperintensity burden. Partial correlations with clinical and cognitive measures were also tested.

Patients with motor neuron disease exhibited significantly lower DTI-ALPS index values relative to healthy controls (*P* = 0.05). Patients with bulbar onset had lower DTI-ALPS values than those with spinal onset (*P* = 0.017). Comparable DTI-ALPS values were found across patients with classical amyotrophic lateral sclerosis clinical presentation and predominant upper or lower motor neuron clinical presentations, with no effect of cognitive diagnosis or genetic status. DTI-ALPS exhibited a significant correlation with disease duration (r = −0.38, *P* = 0.01). Motor neuron disease patients presenting insomnia had significantly lower DTI-ALPS values compared to those without sleep disturbances (*P* = 0.002). Significant positive correlations were found between ALPS index and fractional anisotropy values across major white matter tracts, including the internal and external capsules, superior longitudinal fasciculi, anterior, posterior and superior corona radiata, posterior thalamic radiation, fornix and the genu and body of the corpus callosum. This study confirms the presence of altered interstitial fluid diffusivity dynamics across motor neuron disease phenotypes, with greater impairment observed in bulbar-onset cases, patients with longer disease duration, and those experiencing more pronounced sleep disturbances. These findings may support a potential pathogenic role of glymphatic failure in the accumulation of TAR DNA-binding protein 43 proteinopathy and widespread microstructural axonal damage in motor neuron diseases.

## Teaser

Bottale *et al*. report impaired glymphatic function in motor neuron diseases, using Diffusion Tensor Imaging Analysis along the Perivascular Space (DTI-ALPS). Altered perivascular space diffusivity relates to bulbar onset of symptoms, longer disease duration, sleep problems and white-matter damage, suggesting a role for this system in disease progression and neurodegeneration.

## Graphical Abstract

**Graphical Abstract:**
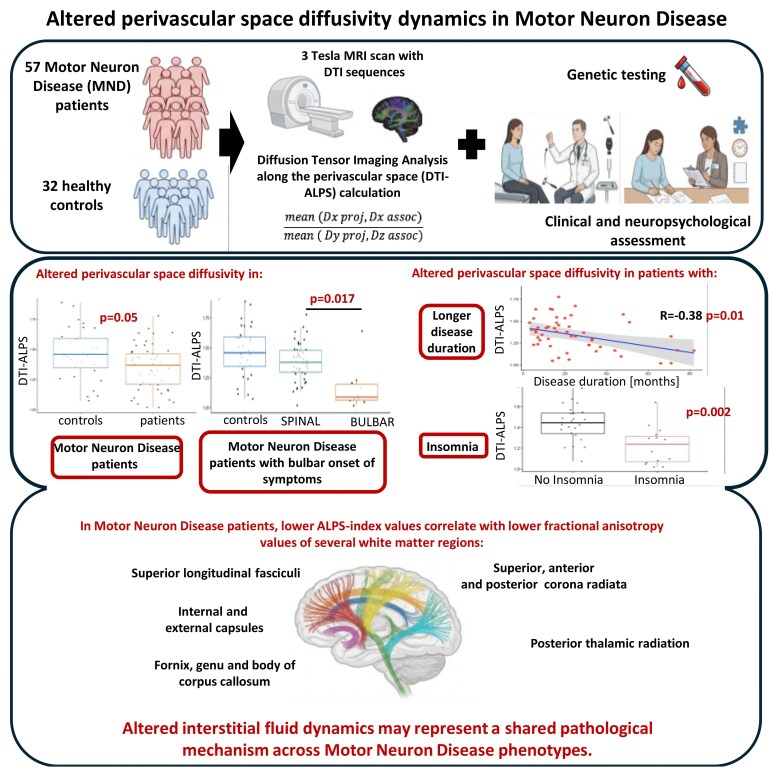

## Introduction

Amyotrophic lateral sclerosis (ALS) is a progressive neurodegenerative disorder characterized by the involvement of both upper (UMN) and lower motor neurons (LMN), ultimately leading to muscle weakness, atrophy and paralysis. ALS pathophysiology is highly complex and multifactorial, reflecting the interplay of genetic, molecular, environmental and immune contributors. Major biological pathways include RNA metabolism dysregulation, impaired proteostasis, mitochondrial dysfunction, oxidative stress and disrupted axonal transport. Approximately 10% of ALS cases are familial and are most frequently linked to mutations in *C9orf72, SOD1, TARDBP* and *FUS* genes, with *C9orf72* hexanucleotide repeat expansions representing the leading genetic cause. These alterations converge on TDP-43 protein aggregation, ultimately driving neuronal injury.^1,2^ In parallel, cerebrospinal fluid (CSF) concentrations of pro-inflammatory cytokines and oxidative stress markers reflect and may further sustain the toxic microenvironment characteristic of ALS.^2^

Despite these advances, the pathophysiology of ALS remains only partially elucidated. Emerging evidence suggests that the glymphatic system, a brain-wide clearance pathway mediated by astrocytes, may contribute to the maintenance of brain homeostasis. This system facilitates the removal of neurotoxic substances by allowing CSF to enter the brain through arterial perivascular spaces, with aquaporin-4 (AQP4) channels expressed on astrocytic endfeet driving the exchange between CSF and interstitial fluid.^2-5^ Metabolic waste products are subsequently conveyed along venous perivascular pathways towards meningeal lymphatic vessels for drainage. In vivo imaging techniques, including intrathecal contrast-enhanced MRI and dynamic PET, have enabled the study of CSF clearance and glymphatic flow, highlighting the relevance of this system to brain health and suggesting that its dysfunction may be involved in pathological aggregate accumulation across CNS proteinopathies.^6^

Diffusion tensor imaging analysis along the perivascular spaces (DTI-ALPS) is a non-invasive and easily accessible method that has been recently proposed for evaluating interstitial fluid diffusivity dynamics in several neuroinflammatory and neurodegenerative conditions.^7-9^

Although the DTI-ALPS index does not provide a direct measure of whole-brain glymphatic function, it strongly correlates with glymphatic clearance following intrathecal gadolinium administration,^10^ making it a valuable proxy for local brain metabolic clearance.^11,12^ Recent research has increasingly pointed to lower DTI-ALPS indexes in patients with ALS, although correlations with motor disability and sleep disturbances are debated^13,14^ and this impairment does not fully differentiate ALS from other neurodegenerative diseases like Parkinson’s disease.^15^ A recent—although small-sized—longitudinal study suggested persistent glymphatic impairment in ALS, compared to primary lateral sclerosis (PLS) patients and healthy controls, suggesting that glymphatic dysfunction may vary across MND phenotypes, reflecting different underlying pathological mechanisms.^14^ The relationship of DTI-ALPS with cognitive or genetic status in MND has not been specifically addressed in the previous literature.

This study aims to investigate interstitial fluid diffusivity dynamics across MND phenotypes employing the DTI-ALPS index as a non-invasive imaging biomarker in a well-characterized cohort of patients, exploring its clinical correlates and relationship with white matter (WM) damage as measured by conventional DTI metrics. Through these objectives, we aim to elucidate the pathophysiological relevance of perivascular space diffusivity as a proxy of glymphatic alterations in MND and assess their potential diagnostic and prognostic value.

## Materials and methods

### Participants

In this retrospective study, we included 64 patients with MND who were referred to IRCCS Ospedale San Raffaele in Milan between June 2017 and June 2023 for brain MRI acquisition on a 3 Tesla scanner, following a standardized neuroimaging protocol. All patients had a confirmed clinical diagnosis of possible, probable or definite ALS according to the El Escorial Criteria.^16^ Two patients were excluded because *SOD1* mutated, as these cases are known to lack TDP-43 pathology, thereby ensuring a more homogeneous pathological substrate within the study cohort. After reviewing MR images, four patients were excluded due to a high cerebrovascular burden or motion artefacts and one because some MRI sequences went missing. Therefore, 57 patients were included (42 with ALS, 7 with predominant LMN [PLMN] involvement and 8 with predominant UMN involvement [PUMN]).

A group of 32 healthy controls, age- and sex-matched to the patient cohort, was also recruited. Controls were included if they had a normal neurological assessment, a Mini-Mental State Examination (MMSE) score of 28 or higher and no family history of neurodegenerative diseases.

All subjects had to meet the following inclusion requirements:

- Be right-handed;^17^
- Have no significant medical conditions, especially major cardiovascular disorders such as end-stage heart failure or atrial fibrillation based on clinical history, that may affect glymphatic flux;
- Have no history of substance misuse;
- Have no major neurological, psychiatric, or systemic disorders;
- And have no conditions that would prevent them from receiving an MRI.

Table 1 summarizes the demographic and clinical characteristics of patients with MND and healthy controls.

**Table 1:** Demographic and clinical characteristics

	HC	MND	*P*
*N*	32	57	-
Age	62.15 ± 8.56 (40.72–75.69)	62.26 ± 11.53 (24.14–80.56)	0.61
Sex [M/F]	17/15	34/23	0.35
Education [years]	11.62 ± 4.14 (5–20)	11.7 ± 3.61 (5–18)	0.97
Spinal/bulbar onset	-	47/10	-
Classic ALS/PUMN/PLMN	-	42/8/7	-
Disease duration at MRI [mo]	-	22.7 ± 18.9 (3–82)	-
ALSFRS-r at MRI	-	40.69 ± 4.1 (31–47)	-
UMN score	-	6.9 ± 5.25 (0–16)	-
King’s stage (1/2/3/4)	-	9/20/7/1	-
Genetic mutations (*C9orf72*/*NEK1*/none)	-	4/1/52	-

Data are reported as mean ± standard deviation (range). *P*-values were derived from ANOVA models followed by Bonferroni-corrected post hoc pairwise comparisons. Statistical significance was set at *P* < 0.05.

Abbreviations: ALS, amyotrophic lateral sclerosis; ALSFRS-r, ALS Functional Rating Scale Revised; HC, healthy controls; MMSE, Mini-Mental State Examination; MND, motor neuron disease; mo, months; PLMN, primary lower motor neuron; PUMN, primary upper motor neuron; UMN Score, upper motor neuron score.

Prior to participation and MRI acquisition, each participant provided written informed consent, and the study was approved by the local ethical commission. The Declaration of Helsinki and appropriate clinical standards were followed in this investigation.

### Clinical evaluation

A group of experienced neurologists carried out the clinical evaluation while blind to MRI results. King's ALS clinical staging,^18^ the revised version of ALS Functional Rating Scale (ALSFRS-r)^19^ and UMN score^20^ were utilized to determine the level of disease severity. For the self-reported evaluation of sleep quality, the NPI^21^ and Hamilton Rating Scale for Depression (HDRS)^22^ specific items were used. Disease duration was calculated as the interval, expressed in months, from reported symptom onset specified in clinical records to the date of MRI acquisition. This information was missing for seven patients.

### Neuropsychological assessment

A comprehensive cognitive evaluation was administered to patients and controls by experienced neuropsychologists blinded to MRI results. The standardized and validated tests employed in the assessment are detailed in Supplementary Table 1. The assessment covered the following cognitive domains:

- Global cognition: Mini-Mental State Examination (MMSE);^23^
- Memory: Rey Auditory Verbal Learning Test—immediate, delayed recall and recognition (RAVLT),^24^ and digit span forward;^25^
- Attention and executive functions: Attentive matrices,^26^ Digit span backward,^27^ Trail making test;^28^
- Visuospatial abilities: Rey–Osterrieth complex figure copy;^29^
- Language: Token test;^30^
- Fluency: Phonemic and semantic fluency tests,^31^
- Behaviour and mood: NPI,^21^ Frontal Behavioural Inventory (FBI),^32^ ALS Frontotemporal dementia Questionnaire (ALSFTD-Q)^33^ and HDRS;^22^
- ALS-specific cognitive and behavioural profile: ECAS—Edinburgh Cognitive and Behavioural ALS Screen.^34^

Cognitive and behavioural status was defined based on performance across cognitive domains and on the presence of behavioural changes, in accordance with the revised consensus criteria for the diagnosis of frontotemporal dysfunction in ALS (Strong *et al*. 2017).^35^ Based on these criteria, the following neuropsychological phenotypes were defined: cognitive and behavioural normal (MND-cbn), MND with behavioural impairment (MND-bi), MND with cognitive impairment (MND-ci), MND with combined cognitive and behavioural impairment (MND-cibi) and ALS–FTD.

### Genetic screening

Blood samples were collected from all patients. The GGGGCC hexanucleotide repeat expansion in the first intron of *C9orf72* was assessed using a repeat-primed polymerase chain reaction (PCR) assay.^36^ The presence of ≥30 repeats, in association with a characteristic saw-tooth pattern, was considered consistent with a pathogenic expansion. In addition, the coding regions and intron/exon junctions of the *GRN, MAPT, TARDBP, SOD1, FUS, TBK1, TREM2, OPTN*, *NEK1* and *VCP* genes were amplified by PCR using optimized protocols to detect known pathogenic mutations.

### MRI acquisition

MRI analysis was conducted at the Neuroimaging Research Unit, IRCCS San Raffaele Scientific Institute, Milan, by experienced observers, blinded to participants’ identities and diagnostic status. MRI acquisition was performed at the same time point as the clinical and neuropsychological assessments. All participants underwent brain MRI scans on a 3.0 Tesla MRI scanner (Ingenia CX, Philips Medical Systems, Best, The Netherlands) at IRCCS Ospedale San Raffaele, Milan, between 1 p.m. and 7 p.m.^37^ The MRI protocol has been previously described in detail,^38^ and included the following brain sequences for all participants: 3D T1-weighted turbo field echo (TFE); 3D fluid-attenuated inversion recovery (FLAIR); 3D T2; 3D susceptibility weighted image (SWI); and axial pulsed-gradient spin echo (PGSE) single shot DW EPI sequence.

### MRI analysis

#### Preprocessing

Diffusion-weighted data preprocessing comprised skull-stripping, correction for head motion through alignment of all volumes to the first B0 volume and correction for susceptibility-induced field distortions and eddy-current effects, using tools from the FMRIB Software Library (FSL, version 5.0.9). The diffusion tensor was fitted by linear regression using only the low-b shell (b < 1000 s/mm^2^) with FSL’s dtifit, and FA maps were subsequently generated.^38^

#### WM hyperintensity volume quantification

WM hyperintensities (WMH) were manually segmented on FLAIR images using Jim software (version 8; Xinapse Systems, UK). Regions of interest (ROIs) encompassing WMH were delineated slice by slice by trained raters, and total WMH volume was then obtained by summing lesion areas across all slices and multiplying the resulting value by slice thickness.

### Quantification of DTI-ALPS

The quantification of DTI-ALPS indexes was computed using an established pipeline.^38^

For each participant, the 3D FLAIR image and FA colour map were rigidly co-registered to SWI space using the magnitude image of the first SWI echo serving as anatomical reference. Registration was carried out in FLIRT (FSL) using normalized mutual information, and FA maps were resampled into SWI space without contributing to the optimization process.^39^ Visual inspection of all transformations was performed by two experts to verify accurate co-registration and to increase inter-rater reliability.

To accurately localize regions where veins, and consequently perivascular spaces (PVS), follow the x-axis orientation, three contiguous axial slices showing veins perpendicular to the lateral ventricle were selected for each participant, with SWI images used as anatomical reference.

Two cubic ROIs with dimensions of 3 × 3 × 3 mm were delineated on the colour-coded FA map. One ROI (ROI_proj) was delineated over the projection fibre region, whereas the second ROI (ROI_assoc) was delineated over the association fibre region. In the left hemisphere, the primary fibre axis corresponded to the y-axis.

To preserve perpendicularity between the fibre axis and the PVS, ROIs were positioned in the left hemisphere of right-handed patients, where association fibres, particularly the superior longitudinal fasciculus, are sufficiently thick.^12^ We avoided placing ROIs over obviously injured tissue by using as reference the co-registered FLAIR images, and we kept a minimum in-plane distance of 2.5 mm from the edge of FLAIR lesions to further improve accuracy.

After their delineation, the ROIs were registered using the inverse diffusion-to-SWI transformation and linear interpolation to the diffusion imaging space. Because linear interpolation yields fractional values at ROI boundaries, all voxels with values greater than zero—indicating spatial overlap with the original binary ROI—were retained. These voxels were subsequently visually inspected on colour-coded FA maps to confirm their anatomical correspondence to projection (ROI_proj) or association (ROI_assoc) fibre bundles.^40^

Lastly, diffusivity components for each ROI were reconstructed along the x-, y-, and z-axes. The DTI-ALPS index was computed as the ratio between diffusivities oriented parallel to the veins and perpendicular to the fibre bundles (Dx_proj and Dx_assoc) and diffusivities oriented perpendicular to both the fibre bundles and the veins (Dy_proj and Dz_assoc).^7,12^

DTI-ALPS index was computed as follows:


$$DTI-ALPS=\frac{mean(Dx\;proj,Dx\;assoc)}{mean(Dy\;proj,Dz\;assoc)}$$

To assess DTI-ALPS index reproducibility, we conducted both intra- and inter-rater reliability analyses. For this purpose, a random sample of 20 MND patients and 20 healthy controls was selected. Two independent raters (E.G.S. and A.G.) defined the regions of interest for the inter-rater analysis in 10 MND patients and 10 healthy controls and then repeated region placement for the remaining subset of 10 patients and 10 controls 30 days after the initial placement to evaluate intra-rater reliability reducing the possibility of a memory bias. The intraclass correlation coefficient (ICC) was computed, with values above 0.81 classified as ‘almost perfect’.^7^

### TBSS analysis

Voxel-wise diffusion tensor MRI analyses were conducted with tract-based spatial statistics (TBSS) implemented in FSL (version 5.0.9) (http://www.fmrib.ox.ac.uk/fsl/fdt/index.html) to generate skeletonized WM FA maps.

For each participant, FA maps derived from the DWI preprocessing pipeline were non-linearly registered to the FMRIB58 template provided in FSL using FNIRT. A mean FA image was then generated by averaging all aligned FA maps in the common space and subsequently thinned to create a skeleton representing only the WM tracts common to all the participants.^38,41^ Skeletonized FA maps were thresholded to exclude voxels below the intensity of 0.2. Individual FA maps were then projected onto this skeleton, and a 4D WM image containing FA measures from all participants was generated.^38,41^

TBSS was performed to analyse the microstructural integrity of white matter across individuals and correlate FA values with DTI-ALPS.^38,41^

### Statistical analysis

#### Demographic, clinical, neuropsychological and DTI-ALPS data

Demographic and other general clinical parameters were compared between groups using ANCOVA models age-, sex-, and education-adjusted. For post hoc analyses, group differences were examined using estimated marginal means. Bonferroni correction was applied to account for multiple pairwise comparisons, and *P* < 0.05 was adopted as the threshold for statistical significance.

Group comparison analyses of DTI-ALPS indexes (healthy controls versus MND patients, as well as across clinical and cognitive subgroups and genetic status) were performed using ANCOVA models adjusting for age, sex, WMH volume,^42^ and FA values of the area where the ROIs were positioned (i.e. left posterior corona radiata) to control for potential influence of local WM microstructural damage over DTI-ALPS calculation.^43^

Partial correlations were used to test the associations between DTI-ALPS values and measures of motor and cognitive disability. Pearson or Spearman correlation coefficients were applied according to data distribution. All models were adjusted for age, sex, WMH volume and FA of the left posterior corona radiata. Statistical analyses were performed using the R Software, version 4.3.2.

#### TBSS: between-group comparisons

A permutation-based inference method for nonparametric statistical thresholding (‘randomize’, FSL)^44^ was used to perform DT MRI voxelwise statistics. A maximum of 5000 permutations were established.^44^ After correcting for age, FA values were compared between patients with MND and healthy control groups. Threshold-free cluster enhancement (TFCE) was applied to correct for multiple comparisons at the cluster level. Between-group differences were considered statistically significant at *P* < 0.05.

#### TBSS: correlations between DTI-ALPS and DTI values

Regression models were performed in FSL in order to determine whether FA values were correlated to DTI-ALPS at a voxelwise level. The TFCE option was used to adjust for multiple comparisons at the cluster level and evaluate the results at *P* < 0.05.

## Results

### Clinical, genetic and neuropsychological findings

Study groups were comparable in terms of age, sex and education (Table 1). Among MND patients, 47 presented a spinal onset of symptoms, while 10 patients had a bulbar presentation. In terms of clinical phenotype, 42 had a classic ALS presentation, 7 had a predominant lower motor neuron (PLMN) presentation and 8 presented with predominant upper motor neuron (PUMN) signs.^45^ Genetic screening identified a pathogenic *C9orf72* expansion in four patients while one patient had a *NEK1* mutation. Clinical and genetic data are shown in Table 1.

Regarding neuropsychological findings, after adjusting for age, sex and education, a statistically significant difference was found in MMSE score between healthy controls and MND groups. Additional significant differences were observed in semantic and phonemic fluencies, executive (including digit span backward and Trail Making Test), language comprehension (Token Test) and memory measures (RAVLT recall). Applying strong revised criteria,^35^ MND patients were assigned to the following neuropsychological categories: 29 MND-cbn, 5 MND-bi, 7 with MND-ci, 6 MND-cibi and 4 with a diagnosis of ALS-FTD. Six patients could not be classified, due to insufficient cognitive data.

Based on the score of the insomnia items of the HDRS (Initial Insomnia, Central Insomnia, Terminal Insomnia) and/or NPI, MND patients were also categorized as with (*n* = 15) or without insomnia (*n* = 24). A score of 0 on all relevant items was considered indicative of no insomnia, whereas a score ≥1 on at least one item led to classification in the insomnia group.

Details regarding neuropsychological data are shown in Supplementary Table 1.

### MRI findings

#### DTI-ALPS index: inter- and intra-rater correlation

Inter-rater reliability analysis yielded mean DTI-ALPS values of 1.36 ± 0.15 for rater E.G.S. and 1.39 ± 0.21 for rater A.G., with an ICC of 0.86. Intra-rater reliability analysis showed an ICC of 0.90, with mean DTI-ALPS values of 1.43 ± 0.20 at the first assessment and 1.46 ± 0.23 at the repeated assessment by the same rater after 30 days.

#### DTI-ALPS: group comparisons

**Figure 1:**
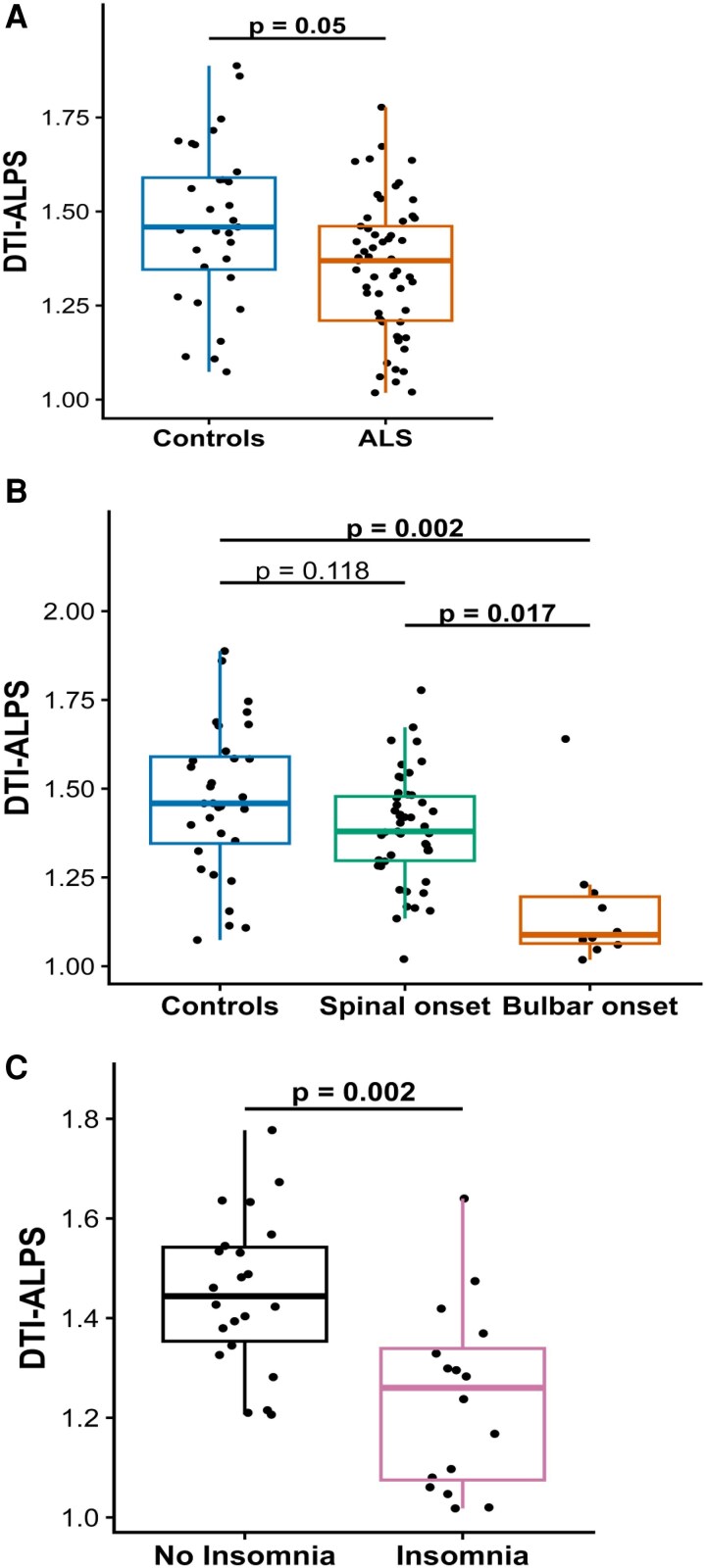
**DTI-ALPS group comparisons**. **(A**) Group comparisons between all patients with MND (*n* = 57) and controls (*n* = 32). (**B**) Group comparisons between controls (*N* = 32) and MND with bulbar (*n* = 10) and spinal onset (*n* = 47). (**C**) Comparison of DTI-ALPS index values between MND patients with (*n* = 15) and without insomnia (*n* = 24). Clinical information about sleep quality was not available for 18 participants. Comparisons between groups were evaluated using ANCOVA models, age-, sex- and FA- and WMH volume-adjusted. Each data point in the figure represents an individual subject. MND, motor neuron disease; WMH, white matter hyperintensity.

Patients with MND exhibited significantly lower DTI-ALPS index values compared to healthy controls (mean ± SD: 1.35 ± 0.18 versus 1.47 ± 0.21; *P* = 0.05), as illustrated in Fig. 1A.

DTI-ALPS index values were significantly lower in MND patients with bulbar symptom onset than in those with spinal onset (mean ± SD: 1.16 ± 0.18 versus 1.38 ± 0.15, *P* = 0.017). Compared to healthy controls, patients with a spinal onset did not show significantly lower values of DTI-ALPS index (*P* = 0.117) (Fig. 1B).

No relevant differences in DTI-ALPS values were observed across MND phenotypes, including classic ALS presentation, PUMN and PLMN. Patients with a classic ALS presentation, however, showed significantly lower DTI-ALPS values compared to healthy controls (mean ± SD: 1.33 ± 0.19 versus 1.47 ± 0.21, *P* = 0.041) (Supplementary Fig. 1A). Similarly, DTI-ALPS values did not differ across cognitive diagnostic subgroups (Supplementary Fig. 1B) or between MND patient groups divided into genetic versus sporadic cases (Supplementary Fig. 1C).

ALPS index values were observed to be significantly lower in MND patients with insomnia than in those without sleep disturbances (mean ± SD: 1.22 ± 0.18 versus 1.44 ± 0.17, *P* = 0.002) (Fig. 1C).

#### TBSS: between-group comparisons

**Figure 2:**
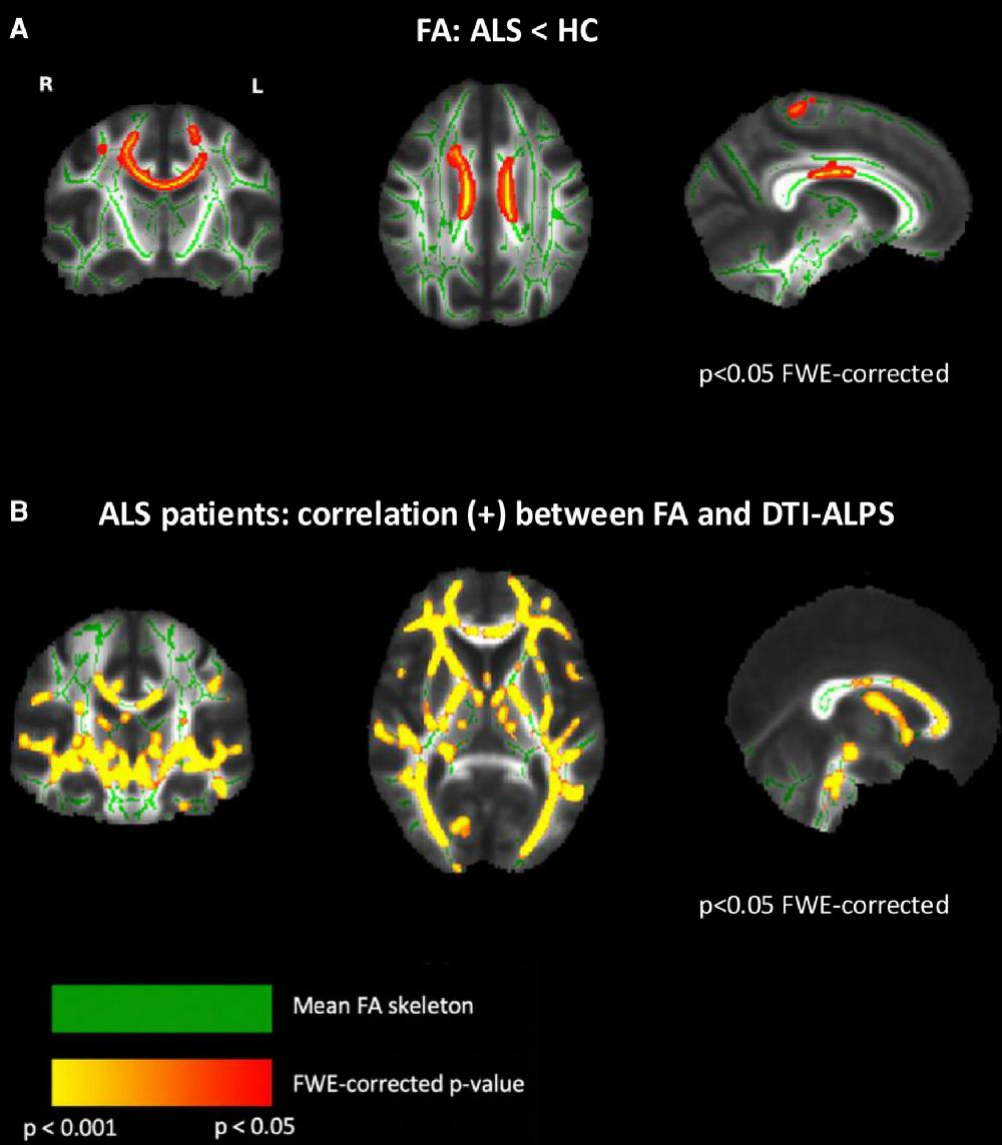
**TBSS analyses: FA comparisons and correlations**. (**A**) Regions with significantly higher FA values in controls (*N* = 32) compared to MND patients (*N* = 57). Voxel-wise group comparisons were performed using a general linear model with non-parametric permutation testing and Threshold-Free Cluster Enhancement, adjusting for age. (**B**) Regions showing a significant positive correlation between DTI-ALPS and FA values considering MND patients only (*N* = 57). Voxel-wise correlations between DTI-ALPS and FA were performed using a general linear model with non-parametric permutation testing and Threshold-Free Cluster Enhancement. Only significant correlations are reported (*P* < 0.05, FWE-corrected). FA, fractional anisotropy; FWE, family wise error; HC, healthy controls; MND, motor neuron disease; TBSS, tract-based spatial statistics; WMH, white matter hyperintensity.

Relative to healthy controls, patients with MND presented with significantly lower FA values in bilateral superior corona radiata, the body of the corpus callosum and right superior longitudinal fasciculus (Fig. 2A).

### Correlation analyses

#### Clinical correlations

**Figure 3:**
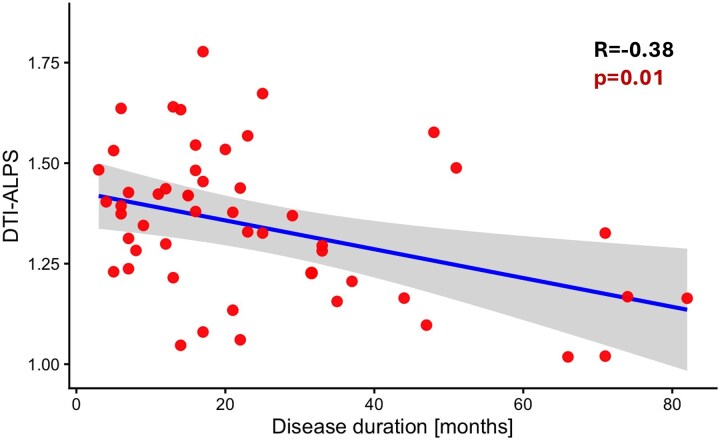
**DTI-ALPS correlation with disease duration**. Correlations with clinical measures were tested using partial correlations, age-, sex-, FA and WMH volume- adjusted. Spearman rank correlation coefficient (R) and *P* value (*P*) are shown. Clinical information about disease duration was not available for seven out of 57 patients. Each data point in the figure represents an individual subject. FA, fractional anisotropy; WMH, white matter hyperintensity.

In patients with MND, DTI-ALPS index values were significantly negatively correlated with disease duration after adjustment for age, sex, FA and WMH volume (r = −0.38; *P* = 0.01) (Fig. 3).

No significant correlations were observed for clinical scales measuring disease severity (i.e. ALSFRS-r, King's ALS clinical staging, UMN Score).

#### Neuropsychological correlations

No statistically significant associations were observed between DTI-ALPS indexes and MMSE, ECAS or any other cognitive score of MND patients.

#### TBSS: relationships between WM microstructural damage and DTI-ALPS

In MND patients, DTI-ALPS index values were significantly positively correlated with FA values of extensive, bilateral symmetric WM regions (Fig. 2B; *P* < 0.05, FWE-corrected). The regions predominantly involved were the anterior and posterior limbs of the internal capsules, the external capsules, the superior longitudinal fasciculi, the anterior, posterior and superior corona radiata and the posterior thalamic radiation bilaterally, and medially the fornix, the body and genu of the corpus callosum.

## Discussion

Using the DTI-ALPS approach, our study contributes to the expanding body of evidence suggesting altered perivascular space diffusivity dynamics in patients with MND. While a recent study demonstrated lower DTI-ALPS values as a suggested marker of glymphatic impairment in early-stage ALS,^13^ we extend these findings by showing that lower DTI-ALPS values are observed across the MND spectrum and are associated with longer disease duration. These findings are also consistent with other recent studies, which reported similar results in ALS patients.^14,15^ Moreover, our study introduces two additional aspects: (i) a systematic integration of clinical, genetic and neuroimaging markers of WM microstructural damage to provide a comprehensive characterization of the patient cohort and (ii) the observation of potential associations between lower DTI-ALPS values, bulbar involvement and sleep disturbances, suggesting a possible pathophysiological bridge linking these core clinical features of MND.

As a first important result, lower DTI-ALPS values in MND patients compared with healthy controls provide further evidence that altered perivascular space diffusivity dynamics may be a feature of MNDs, supporting the concept of an impaired waste-clearance system in these disorders, similar to other CNS proteinopathies underlying neurodegenerative diseases. Impaired glymphatic function has been linked to sleep disturbances in Alzheimer’s and Parkinson’s diseases,^5,46^ although it remains uncertain whether it drives, results from, or merely accompanies these proteinopathies. In the case of MND, it is possible that the early impairment of autonomic function and weakness of bulbar and respiratory muscles might act on the vascular pulsations from the cardiovascular and respiratory systems that influence glymphatic flow.^47^ Consistent with this hypothesis, previous studies showed that AQP4—a water channel expressed in the central nervous system critical for fluid movement across astrocytic endfeet—is overexpressed in ALS,^48^ potentially as a compensatory or protective response. The present study supports such hypothesis by showing lower DTI-ALPS index in patients with a bulbar onset of symptoms. In fact, although previous neuroimaging studies have described distinct structural and microstructural alterations in bulbar-onset ALS—such as early cortical thinning and frontotemporal involvement^15^—direct evidence linking this phenotype to altered perivascular space diffusivity dynamics as a proxy of glymphatic dysfunction is missing. Our observations reinforce the idea of an early impairment of clearance mechanisms, possibly linked to faster protein accumulation and impaired sleep-dependent clearance in MND patients with a bulbar onset of symptoms. Considering that this clinical presentation is related to a worse prognosis,^49^ we hypothesize that a bulbar onset of symptoms could serve as a marker of more profound altered interstitial fluid diffusivity, with implications for prognosis and personalized disease monitoring. The novelty and consistency of this observation highlight the importance of further exploring the mechanistic links between bulbar presentation, disease aggressiveness and glymphatic failure in future longitudinal studies.

In our cohort, MND patients reporting sleep disturbances—measured via validated clinical tools such as the NPI and HDRS—showed significantly lower DTI-ALPS index values compared to those without insomnia. This finding supports and extends prior evidence from non-ALS populations linking poor sleep quality to glymphatic dysfunction,^50,51^ and reinforces the concept of sleep as a modulator of glymphatic clearance in MND and other neurodegenerative diseases. Physiologically, the glymphatic system is most active during slow-wave sleep, a phase that facilitates the clearance of neurotoxic proteins including TDP-43, which are central to ALS pathology.^51^ In ALS, sleep is often fragmented by nocturnal hypoventilation, increased respiratory effort and reduced central respiratory drive—all of which may impair this essential clearance mechanism. Our findings suggest that such disturbances are not just symptomatic consequences of disease but may actively contribute to pathological burden via impaired glymphatic flow. Despite its relevance, the interaction between sleep and glymphatic function remains under-investigated in ALS.^5^ Our data contribute novel evidence to support the existence of a sleep-glymphatic axis in MND, with potential clinical implications. Therapeutic strategies aimed at improving sleep quality—such as optimization of ventilation, early respiratory support, or pharmacological modulation of sleep—could help preserve glymphatic efficiency and potentially slow neurodegeneration in ALS.

While a recent study focused exclusively on early-stage ALS patients with relatively preserved function,^13^ our cohort included individuals with longer disease duration yet consistently showed lower DTI-ALPS values. We also showed a significant negative correlation between DTI-ALPS values and disease duration. This indicates that alterations in perivascular fluid diffusivity are not limited to the initial stages of disease but persist—and possibly, even worsen—as neurodegeneration progresses, potentially reflecting a progressive congestion of glymphatic flow as pathological burden accumulates. In line with this, a recent longitudinal study reported a stable impairment of DTI-ALPS in ALS patients over time, while no changes were observed in PLS, although the follow-up sample size was small for both groups.^14^

Our findings partially diverge from the few previous reports in ALS, which either did not observe significant associations between the ALPS index and clinical parameters^14^ or reported an association with clinical disability.^13^ In our cohort, the association between the ALPS index and disease severity scores did not reach statistical significance, whereas a relationship with disease duration was observed. Such discrepancies may stem from differences in sample size or disease stage distribution. Importantly, clinical severity scales capture motor disability at a single time point and may not linearly reflect cumulative neurodegenerative or microstructural changes, whereas disease duration more closely represents long-term exposure to disease-related pathology.^52,53^

In addition, we did not observe any correlation with genetic status. This association has not been extensively investigated in prior studies—which, similarly, do not report it—and our cohort included only a small number of patients carrying mutations (4 *C9orf72* and 1 *NEK1*). Larger samples including a greater number of patients carrying a *C9orf72* pathogenic expansion are needed to verify possible divergence in mechanisms leading to glymphatic overload due to dipeptide accumulation, in addition to TDP-43.

Furthermore, the absence of significant associations with cognitive scores contrasts with some previous reports in other neurodegenerative conditions, such as Alzheimer’s disease (AD).^12^ While correlations between DTI-ALPS and cognition have been described in the literature, their presence and strength vary according to disease population, cognitive domains assessed and sample size. In MND, cognitive impairment is often more heterogeneous and less prominent than AD, which may reduce the sensitivity of cross-sectional cognitive measures to detect such associations. Taken together, our findings suggest that altered interstitial fluid diffusivity dynamics may be relatively independent of the purely cognitive aspects of MND.

TBSS analysis revealed significantly lower FA values in ALS patients compared to healthy controls, particularly in key white matter tracts such as the body of the corpus callosum, superior longitudinal fasciculus and corona radiata. These findings are consistent with previous studies highlighting microstructural damage in corticospinal and associative fibres in ALS, which constitute the signature of white matter microstructural damage in MND.^54^ Importantly, we found a significant positive correlation between DTI-ALPS values and FA values across widespread white matter regions. This suggests that dysfunctional perivascular fluid diffusivity may coexist with—and potentially contribute to—axonal degeneration. A less efficient glymphatic system may, in fact, impair the clearance of neurotoxic waste products such as TDP-43, exacerbating the pathogenic cascade and promoting neuronal demise. Together, these data may support the hypothesis that altered interstitial fluid diffusivity dynamics and white matter microstructural damage are mechanistically linked. However, given the cross-sectional design of our study, no conclusions can be drawn regarding the temporal or causal relationship between these imaging measures.

Key limitations of this study are its cross-sectional design and the relatively small sample size. Beyond these study-specific aspects, the DTI-ALPS method itself presents intrinsic limitations. In fact, although widely used in several previous reports to infer glymphatic function,^13-15^ the ALPS index primarily reflects the directional diffusivity of water along perivascular spaces and does not capture the full complexity of the glymphatic system. Therefore, as highlighted in a recent review,^11^ its interpretation requires caution, as it may also be influenced by anatomical variability and technical factors (including the need for several manually guided steps, which may introduce operator-dependent variability). Nevertheless, the method has demonstrated significant correlations with more established—though invasive—techniques for studying glymphatic flow such as glymphatic MRI after intrathecal gadolinium administration, where DTI-ALPS showed strongly significant inverse correlations with the percentage change in signal unit ratio (SUR) between baseline and 39 h after intrathecal gadolinium administration (Pearson’s r = −0.77 to −0.84, *P* < 0.001) in a cohort of 39 patients with a wide range of neurological conditions.^10^

Taken together, these considerations suggest that further work is warranted to clarify the longitudinal stability and clinical relevance of the DTI-ALPS index. Future studies adopting multimodal designs, including sleep assessments and complementary biomarkers, may help refine its interpretation and better delineate the potential contribution of glymphatic dysfunction to MND pathophysiology. Finally, although the present study employed a manually guided DTI-ALPS approach, future studies should assess the robustness and reproducibility of these findings using fully automated pipelines.

Within these constraints, our findings are consistent with the hypothesis that glymphatic alterations may be involved in ALS. Interventions aimed at preserving or restoring glymphatic function—through sleep optimization, respiratory support, or modulation of AQP4 expression—could represent a novel therapeutic approach to delay disease progression and reduce global neurological burden. Notably, despite its limitations, the non-invasive and easily implementable nature of DTI-ALPS supports further investigation of its potential utility as a biomarker for tracking disease progression or evaluating therapeutic effects in MND and other neurodegenerative conditions.

## Supplementary Material

fcag307_Supplementary_Data

## Data Availability

Due to privacy and ethical restrictions, the dataset generated and analysed during the current study cannot be made publicly available. Access to the data may be granted by the corresponding author upon reasonable request.
