# Supplementary material for: Altered perivascular space diffusivity dynamics in motor neuron disease
Source: Brain Commun. 2026 Aug 10;8(4):fcag307. doi: 10.1093/braincomms/fcag307 (PMC13479384; doi:10.1093/braincomms/fcag307)
Supplement: Supplementary file 1 [file fcag307_supplementary_data.pdf]

Supplementary Materials

Supplementary Table 1. Neuropsychological assessment

	Healthy Controls	MND patients	p-value
**Global cognition**			
**MMSE**	29.31 ± 0.86 (28-30)	28.1±2.07 (22-30)	**0.005**
**Memory**			
**Digit span, forward**	6.21±1.18 (4-8)	5.62±1.28 (3-9)	0.06
**Rey's figure recall**	5.07±1.3 (3-8)	4.22±1.4 (0-9)	0.942
**RAVLT, immediate recall**	51.45 ± 8.29 (40-72)	39.8 ± 11.64 (15-65)	**<0.001**
**RAVLT, delayed recall**	11.17 ± 2.28 (7-15)	8.12 ± 3.34 (0-15)	**<0.001**
**RAVLT, recognition**	14.6±0.6 (13-15)	13.8±1.9 (7-15)	0.108
**Attention and executive functions**			
**Attentive Matrices**	53.36 ± 5.29 (41-60)	50.38 ± 7.84 (40-58)	0.254
**Digit span, backward**	5.07 ± 1.3 (3-8)	4.22 ± 1.4 (0-9)	**0.01**
**Trail Making Test, A**	31.96 ± 7.71 (17.5-48.67)	47.81 ± 22.85 (30-101)	**0.003**
**Trail Making Test, B**	93.33 ± 32.6 (46.73-217)	142.7 ± 124.02 (62-428.38)	0.075
**Trail Making Test, BA**	61.37 ± 29.74 (19.99-181.92)	94.36 ± 114.93 (31-371.1)	0.197
**Visuospatial abilities**			
**Rey figure, Copy**	29.6 ± 3.05 (25-32)	29.81 ± 2.36 (25.5-33)	0.9
**Language**			
**Token Test**	34.17 ± 1.54 (0.5-36)	32.25 ± 1.49 (30-34)	**0.003**
**Fluency**			
**Phonemic Fluency**	39.55 ± 8.17 (27-57)	32.73 ± 13.87 (4-61)	**0.017**
**Semantic Fluency**	50.41 ± 9.76 (34-70)	39.93 ± 11.58 (13-60)	**<0.001**
**Behaviour**			
**FBI**	-	3.52 ± 3.97 (0-16)	-
**ALSFTD-Q**	-	8.55 ± 7.82 (0-31)	-
**NPI**	-	5 ± 4.15 (0-15)	-
**Mood**			
**HDRS**	-	5.38 ± 4.10 (0-19)	-
**ECAS**			
**Language**	-	23.16±4.99 (2-28)	-
**Verbal Fluency**	-	16.67±5.91 (0-22)	-
**Executive**	-	32.38 ± 9.75 (10-46)	-
**ALS-Specific**	-	71.88 ± 17.39 (31-96)	-
**Memory**	-	17.1 ± 8.21 (1-65)	-
**Visuospatial**	-	11.36±1.72 (6-18)	-
**ALS-non-Specific**	-	28.46 ± 9.29 (12-83)	-

**Total score**	-	100.34 ± 20.25 (50-129)	-
**Behavioural Screening**	-	0.95 ± 0.33 (0-3)	-
**Strong's et al classification[1]**			
**ALS-FTD/BI/CI/CI-BI/CBN**	-	4/5/7/6/29	-

Values are means ± standard deviations (range). p-values refer to ANCOVA models adjusted for age, sex and education, followed by post-hoc pairwise comparisons, Bonferroni-corrected for multiple comparisons. The threshold of statistical significance was set at $p<0.05$.

Bold p-values= statistically significant difference between HC and MND patients.

Abbreviations: ALS-FTD: Amyotrophic lateral sclerosis-Frontotemporal Dementia; BI: behavioral impairment; CI: cognitive impairment; ALSFTD-Q: ALS-Frontotemporal Dementia Questionnaire; CIBI: Cognitive and behavioral impairment; CBN: cognitive and behavioural normal; ECAS: Edinburgh Cognitive and Behavioural ALS Screen; FBI: Frontal Behavioural Inventory; HDRS: Hamilton Depression Rating Scale; MMSE = Mini-Mental State Examination; MND: Motor Neuron disease; NPI: Neuropsychiatric Inventory; RAVLT = Rey Auditory Verbal Learning Test.

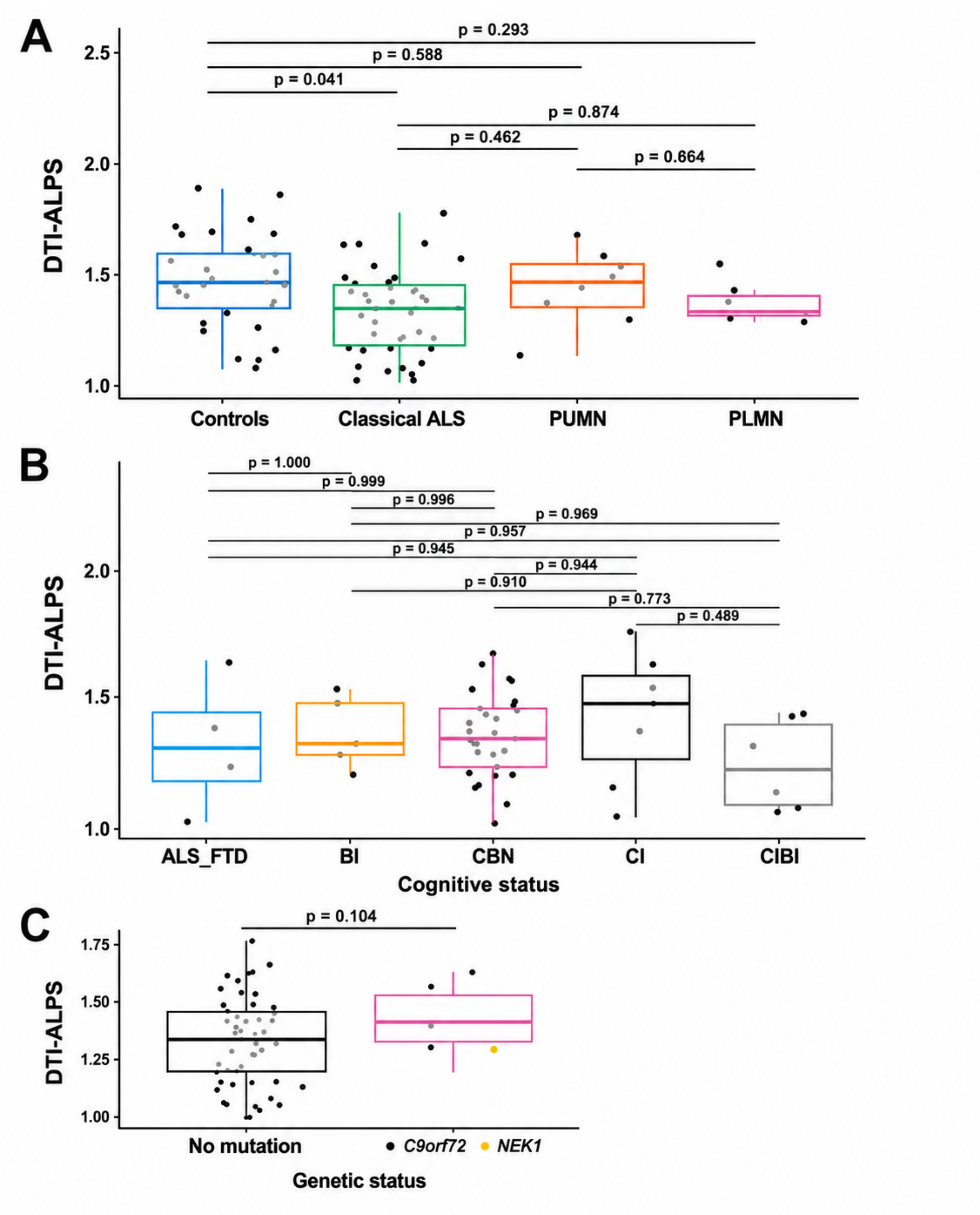

Supplementary Figure 1. Boxplots showing the distribution of DTI-ALPS values across clinical phenotypes, cognitive status, and genetic status. A) Comparison of DTI-ALPS Index between different ALS clinical phenotypes (classic ALS, PUMN, PLMN) and controls. N=89 subjects. B) Comparison of DTI-ALPS Index in ALS patients stratified by cognitive status. N=51 subjects. C) Comparison of DTI-ALPS Index Between Genetic and Sporadic ALS Patients. N= 57 subjects.

Comparisons between groups were evaluated using ANCOVA models, age- sex- FA- and WMH volume-adjusted. Each datapoint represents an individual subject.

Abbreviations: ALS: Amyotrophic Lateral Sclerosis; ALS-FTD: Amyotrophic lateral sclerosis-Frontotemporal Dementia; BI: behavioral impairment; CI: cognitive impairment; CIBI: Cognitive and behavioural impairment; CBN: cognitive and behavioural normal; ECAS: Edinburgh Cognitive and Behavioural ALS Screen; HC: Healthy Controls, PLMN: Primary Lower Motor Neuron; PUMN: Primary Upper Motor Neuron

Supplementary Figure 1. Alt text: Boxplots show diffusion tensor imaging analysis along the perivascular space values across clinical phenotypes, cognitive and behavioural categories, and genetic versus sporadic motor neuron disease subgroups.

References

1. Strong MJ, Abrahams S, Goldstein LH, et al. Amyotrophic lateral sclerosis - frontotemporal spectrum disorder (ALS-FTSD): Revised diagnostic criteria. *Amyotroph Lateral Scler Frontotemporal Degener*. May 2017;18(3-4):153-174. doi:10.1080/21678421.2016.1267768
